# Comparative riverscape genomics of the rainbow darter (*Etheostoma caeruleum*) in glaciated and unglaciated environments

**DOI:** 10.1002/ece3.8422

**Published:** 2021-12-01

**Authors:** Jon M. Luiken, Tony Gamble, Peter B. Berendzen

**Affiliations:** ^1^ Department of Biology University of Northern Iowa Cedar Falls Iowa USA; ^2^ Department of Biological Sciences Marquette University Milwaukee Wisconsin USA

**Keywords:** Mississippi River, North America, Ozark Plateau, Quaternary, stream hierarchy model, Time lag

## Abstract

Periodic glaciation during the Quaternary period shaped the contemporary riverscape and distribution of freshwater fishes in the Mississippi River drainage of central North America. The rainbow darter (*Etheostoma caeruleum*) is a member of this ichthyofauna and has a disjunct distribution in glaciated and unglaciated environments west of the Mississippi River. Based on glacial history of the region, there are different expectations on the observed spatial genetic structure of populations in these environments. The aim of this study was to utilize genome‐wide SNP data to compare the population genomic structure of the rainbow darter in river networks with disparate glacial histories; the Volga River in the glaciated upper Mississippi River basin and the Meramec River in the unglaciated Ozark Plateau. Individuals were sampled from localities within each river system at distances dictated by the organismal life history and habitat preferences. Riverscape analyses were performed on three datasets: total combined localities of both rivers and one for each river independently. The results revealed a lasting influence of historic glaciation on the population genomic structure of rainbow darter populations. There was evidence of population expansion into the glaciated northern region following glacial retreat. The population genetic signature within the Volga River did not fit expectations of the stream hierarchy model, but revealed a pattern of repeated colonization and extirpation due to cyclic glaciation. The population within the unglaciated Meramec River adhered to the stream hierarchy model, with a directional order of genetic diversity based on the life history and habitat preferences of the species. These results demonstrate the importance of considering the geologic and climatic history of a region as well as the life history of an organism when interpreting spatial genetic patterns.

## Graphical abstract

The aim of this study was to compare the population genomic structure of the rainbow darter in river networks with disparate glacial histories. The results support the hypothesis that historical glacial cycles during the Quaternary period influenced the observed genetic signature of rainbow darter populations in drainages of central North America. They support the prediction that the populations in the glaciated environment in the north does not fit expectations under the stream hierarchy model.

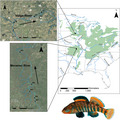

## 1 INTRODUCTION

Freshwater aquatic organisms in fluvial systems have gene flow constraints that are associated with the directional flow of water, habitat heterogeneity, and the dendritic nature of the stream network. Understanding the influences on the evolution of spatial genetic structure of populations in riverine environments is a unique problem that involves a specific set of questions, which are described as riverscape genetics (Davis et al., 2018). Based on the degree of connectedness of the stream network, the distribution of habitat, and life history of the organism, a directional hierarchy in genetic diversity of local populations is expected. This is referred to as the stream hierarchy model (Meffe & Vrijenhoek, 1988). Any modification to the riverscape structure such as dams, loss of habitat, flooding, or alteration of the drainage system can disrupt the patterns of gene flow and lead to changes in the expectations of the population genetic structure (Davis et al., 2018; Meffe & Vrijenhoek, 1988). The goal of this study is to test predictions of the stream hierarchy model of a native freshwater fish distributed in glaciated and unglaciated environments of the upper Mississippi River basin of Central North America.

**FIGURE 1:**
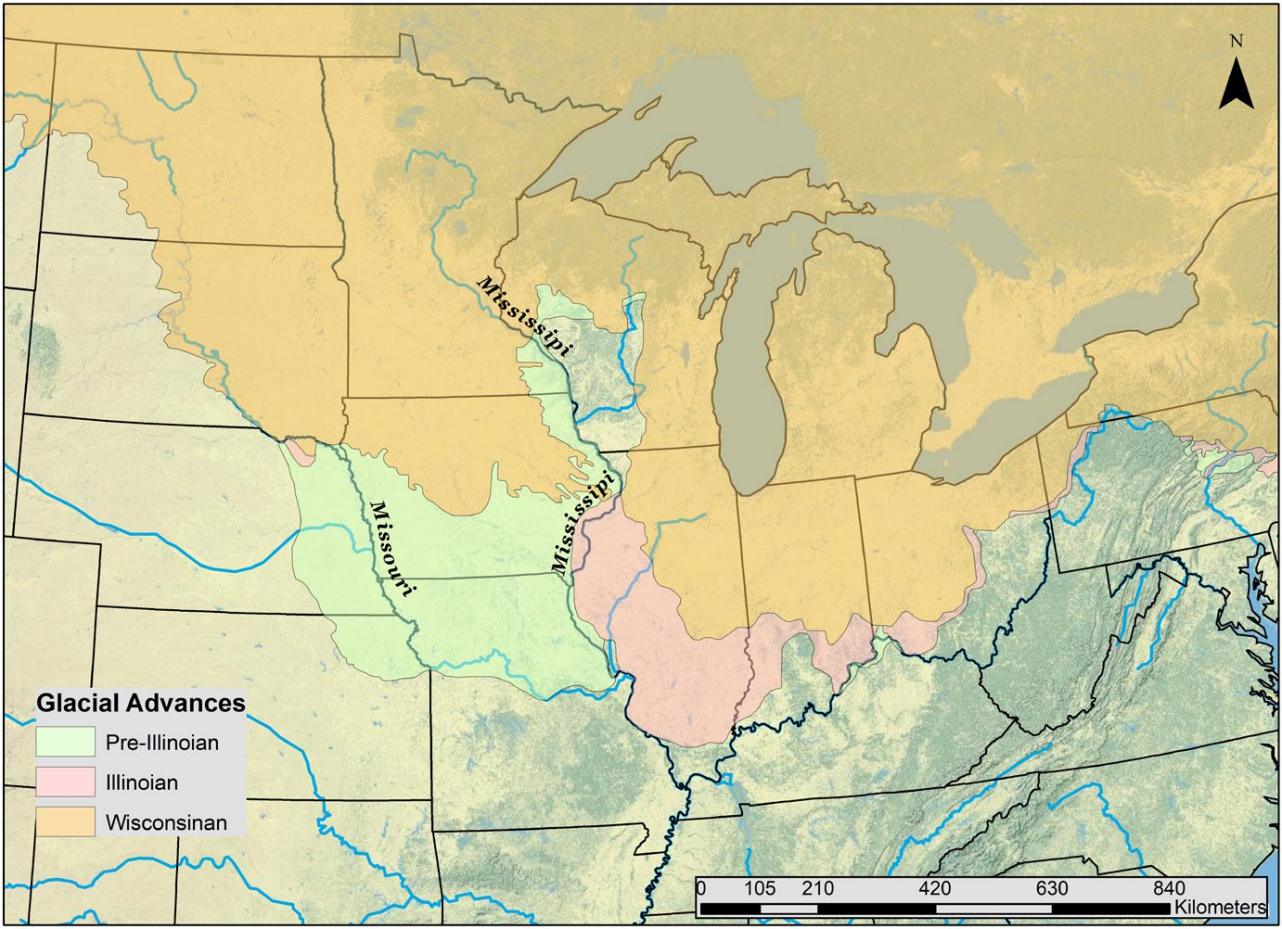
Map of the approximated limits of pre‐Illinoian (2.5–0.5 million years ago), Illinoian (300,000–140,000 years ago), and Wisconsinan (19,000–10,500 years ago) glacial advancements in the central portion of North America

The connectivity of stream networks and the species assemblage present within the upper Mississippi River basin are the result of a complex geologic and climatic history. Recurrent, alternating cycles of glacial advance and retreat by the Laurentide Ice Sheet during the Quaternary period, 2.6 million to 10,500 years ago, reshaped and fragmented the regional landscape and altered drainage patterns and flow regimes (Cupples & Van Arsdale, 2014; Galloway et al., 2011; Knox, 2007; Lowe & Walker, 2014; Figure 1). To the west of the Mississippi River, the southernmost extent of these glacial advances occurred during the Pre‐Illinoian Glacial Stage (2.6–0.3 million years ago), reaching the present‐day Missouri River valley in central Missouri (Burr & Page, 1986; Hobbs, 1999; Thornbury, 1965; Figure 1). The Ozark Plateau and associated stream networks to the south of the Missouri River remained relatively unaffected by the glacial disturbances (Pflieger, 1975).

The distribution of aquatic species in this region was notably affected by the habitat changes in the north, resulting in displacement or extirpation of local populations (Berendzen et al., 2010; Burr & Page, 1986). West of the Mississippi River, the Ozark Plateau provided a refugium for many fish species during the periodic glacial advances of the Quaternary period (Berendzen et al., 2003, 2010; Burr & Page, 1986; Echelle et al., 2014; Near et al., 2001). Following the retreat of the last glacial maximum (19,000 to 10,500 years ago), new dispersal opportunities were created among the newly formed stream connections, which contributed to the expansion of populations northward and the establishment of the contemporary fish assemblage (Berendzen et al., 2010; Burr & Page, 1986; Ray et al., 2006).

**FIGURE 2:**
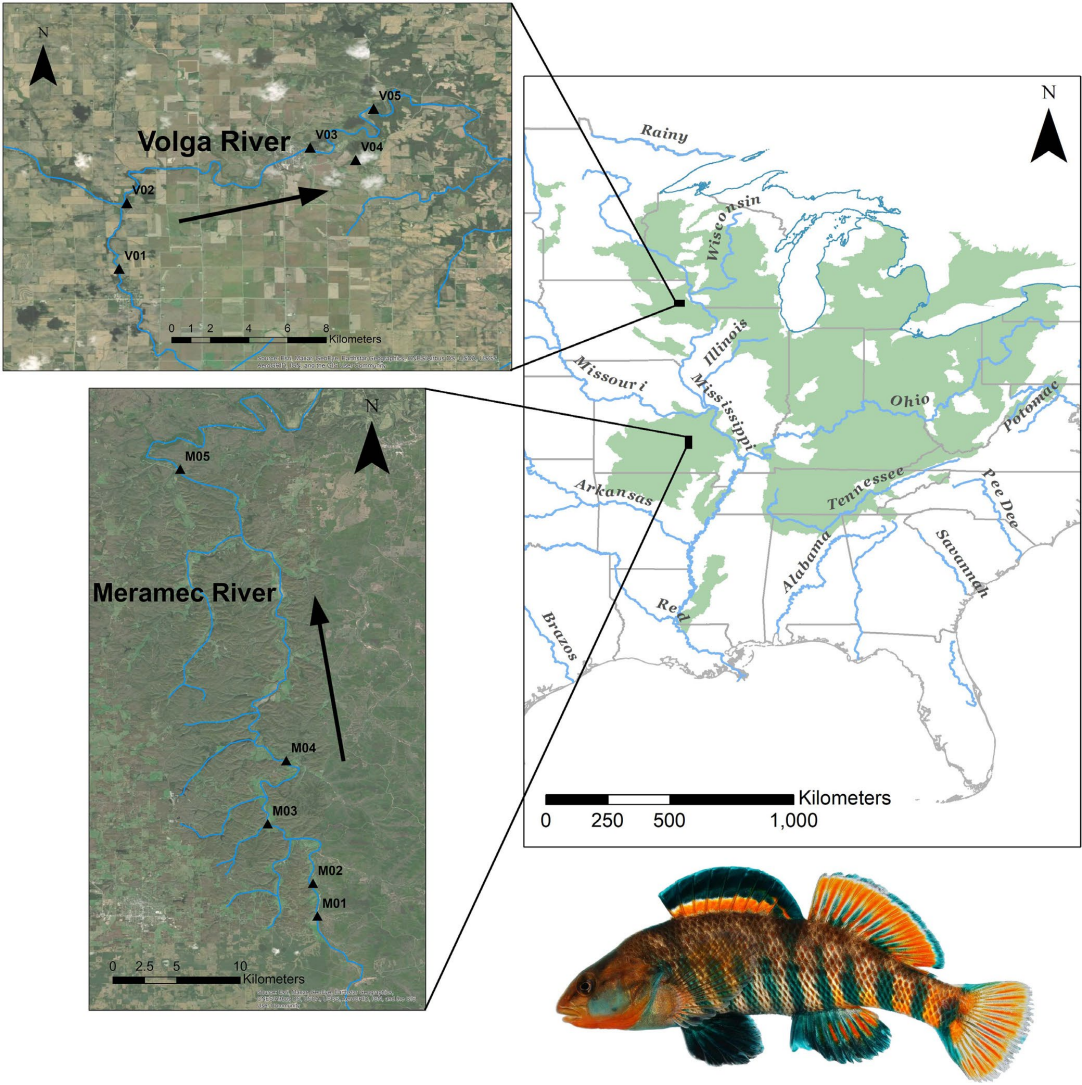
The approximate geographic distribution of the rainbow darter (*Etheostoma caeruleum*) in eastern North America: spatial data obtained from NatureServe (2013). Insets indicate sampling localities within the Volga and Meramec Rivers. Arrows indicate direction of flow of water. Image of a male rainbow darter

*Etheostoma caeruleum*, the rainbow darter, is a member of this assemblage with a broad distribution in small rivers, creeks, and streams in the Mississippi River drainage across eastern North America (Ray et al., 2006; Strange & Burr, 1997; Figure 2). The species is a small, sexually dimorphic fish that displays a preference for shallow, fast‐flowing water within gravel or rubble riffles (Harding et al., 1998; Mueller et al., 2020). Mature individuals exhibit high site fidelity to their natal habitats throughout their life cycle, with breeding males migrating up to 1 km during spring spawning periods (Hicks & Servos, 2017). The rainbow darter is a benthic invertivore that actively forages for a variety of macroinvertebrates that inhabit riffles. Their preferred food sources are much less abundant in the deeper pools separating riffle structures (Mueller et al., 2020). In addition, *E*. *caeruleum* is considered a sentinel species and is relatively sensitive to habitat and water quality changes due to channel siltation, chemical runoff, and related effects of anthropogenic activity (Haponski et al., 2009; Tonnis, 2006).

West of the Mississippi River, *E*. *caeruleum* has a disjunct distribution inhabiting streams in the upper Mississippi River basin and the Ozark Plateau, but is absent in the intervening region (Figure 2). In tributaries of the upper Mississippi River, local populations of the rainbow darter have a patchy, disjunct distribution across the riverscape (Davis et al., 2015). Areas of local concentration are often separated by large river distances. This pattern results from the direct impact of glacial processes on the landscape causing the spatial distribution of suitable habitat to be highly fragmented and heterogeneous. Modern river drainages in this region flow through varying depths of glacial drift overlaying the Tertiary bedrock topography (Burr & Page, 1986; Thornbury, 1965). In contrast, the drainage patterns of the Ozark Plateau have maintained their basic configuration since the late Paleozoic era (Pflieger, 1975). Modern river drainages in this region are characterized by clear, cool high‐gradient streams with course substrate in deeply dissected valleys (Pflieger, 1975; Thornbury, 1965). Local populations of the rainbow darter in this region are widespread and nearly continuous, resulting from the abundant distribution of suitable habitat. The rainbow darter is one of the most abundant fish species in the Ozarks (Pflieger, 1975).

In this study we compare the fine‐scale population genetic structure of rainbow darter populations in two similar river systems, one located in the glaciated environment of the upper Mississippi River basin and one in the unglaciated environment of the Ozark Plateau. We test the hypothesis that climatic and geologic processes during the Quaternary period influenced the observed genetic signature and disrupted expectations under riverscape genetic models. Under the stream hierarchy model, the population genetic structure is consistent with the structure of the stream network, distance between local populations, connectedness of the habitat and life history of the organism (Brauer et al., 2018; Meffe & Vrijenhoek, 1988). Since the rainbow darter exhibits high site fidelity and short dispersal distances, the expectation is that local populations will be partially isolated with limited gene flow and evidence of isolation by distance in an upstream‐to‐downstream pattern. We predict that the population genetic structure of *E*. *caeruleum* in the glaciated environment in the North will not fit expectations under the stream hierarchy model and will exhibit low levels of genetic diversity. Conversely, we predict that the population genetic structure of *E*. *caeruleum* in the unglaciated environment in the South will fit expectations under the stream hierarchy model and exhibit higher levels of genetic diversity. In this study, we utilize genome‐wide SNPs generated using RADseq to infer fine‐scale population genetic structure in each river system.

## 2 METHODS

### 2.1 Study region and sampling methods

The Volga (Fayette County, IA) and Meramec (Crawford/Dent Counties, MO) rivers were selected as systems for comparison. Both waterways are historically known to contain abundant resident populations of *E*. *caeruleum* and have segments that are relatively undisturbed and unimpeded (Davis et al., 2015; Ray et al., 2006). The Volga River is a tributary of the upper Mississippi River within the previously glaciated region of North Central North America. The Meramec River, in contrast, is a tributary of the Mississippi River traversing land within the historically unglaciated Ozark Plateau. Both systems are located to the west of the Mississippi River (Figure 2).

Local populations in each river system were sampled from riffle sites separated by at least 2 km of river distance (Figure 2; Table S1). This distance was chosen because of the notable high site fidelity exhibited by *E*. *caeruleum* individuals (Hicks & Servos, 2017). Candidate segments and sampling localities in both rivers were identified using freely available GIS data in combination with satellite imagery from Google Earth. This was done to ensure adequate accessibility to each location and a lack of artificial barriers such as low‐head dams within the sampling areas. A total of 10 *E*. *caeruleum* individuals were collected from each locality using kick seining techniques and backpack electrofishing where permitted. This sampling strategy was chosen because it has been shown that fewer individuals are necessary to detect fine‐scale population genetic structure when analyzing large genome‐wide SNP datasets due to the large number of high quality of loci that are sampled (Jeffries et al., 2016; Sunde et al., 2020). Pectoral fin tissue was taken from the right side of positively identified fish and stored in 95% ethanol for DNA work; whole specimens were preserved in formalin as vouchers and deposited at the Bell Museum of Natural History Fish collection, University of Minnesota (Table S1).

### 2.2 DNA sequencing and SNP genotyping

A reduced representation genomic dataset using RADseq was generated to genotype all individuals using genome‐wide SNPs. RADseq libraries were prepared using a protocol modified from Etter et al. (2012), as specified in Gamble et al. (2015). Genomic DNA was extracted from ethanol preserved pectoral fin clips using a DNeasy Blood and Tissue Kit (Qiagen). Approximately 1 μg of DNA from each sample was digested at 37℃ for 2 h using the high‐fidelity *SbfI* restriction enzyme (New England Biolabs). A two‐adapter process was utilized to create DNA libraries from the digested samples, with 10–15 individually barcoded samples allocated to each Illumina library pool. P1 adapters with unique barcodes were ligated to the specific *SbfI* overhang sequence exposed during the digestion process. Samples were then pooled, sheared using a sequence of cyclic sonication (Diagenode Bioruptor), and size selected into approximately 300–400 base pair (bp) lengths using magnetic beads in PEG/NaCl buffer (Rohland & Reich, 2012). The libraries were blunt‐end repaired and dA tailed. P2 adapters were then ligated to each pooled library to provide unique Illumina identifiers and additional barcode sequences necessary for the Illumina sequencing process (Andrews et al., 2016). All libraries were amplified via PCR using Q5 DNA polymerase (New England Biolabs) for 14 cycles, cleaned, and size selected an additional time using a GeneRead Size Selection Kit (Qiagen). Genomic libraries were quantified using a Qubit fluorometer and pooled for Illumina sequencing. DNA fragment sizes were verified using a Bioanalyzer assay (Agilent) and the pooled libraries were sequenced with paired‐end 75 and 100 bp reads on Illumina HiSeq 4000 (75 bp paired‐end) and NovaSeq 6000 (100 bp paired‐end) platforms at the Genomics Division of the Iowa Institute of Human Genetics, University of Iowa.

Raw Illumina sequence data were demultiplexed and filtered by their adapter sequences using the *process_radtags* command in *STACKS v*.*2*.*54* (Catchen et al., 2013). Two mismatches were allowed within the adapter sequences ‘‐‐*adapter_mm 2*’ and any reads marked as failures by Illumina's purity filter were discarded by using the ‘‐‐*filter_illumina*’ options. The program flags, ‘‐*c*’, ‘‐*q*’, and ‘‐*r*’ were utilized to remove data with uncalled bases, low phred33 quality scores, and recover and repair barcode segments (Rochette et al., 2019). Read lengths for all samples were trimmed to a 70 bp length to provide data continuity between both Illumina sequencing platforms and to remove any low‐quality bases from the 3’ end of reads (Catchen et al., 2011). Files output from *process_radtags* were then concatenated to a single file named for each respective sample (Rochette & Catchen, 2017).

Due to the lack of an available reference genome for *E*. *caeruleum*, the *denovo_map*.*pl* script (*STACKS*) was run to create a consensus sequence catalog, generate SNP loci, and formulate candidate alleles for individual fish (Catchen et al., 2011). Parameters for the assembly process were optimized using testing procedures described in Paris et al. (2017). The minimum depth of coverage required to create a stack, a set of short reads which match exactly, was set to a value of 3 ‘‐*m 3*’. The maximum distance allowed between stacks was set to 3 nucleotides ‘‐*M 3*’ while the mismatches allowed between sample loci during catalog sequence assembly was set at 2 ‘‐*n 2*’. The ‘‐‐*rm*‐*pcr*‐*duplicates*’ program flag was also applied to reduce the effect of PCR amplification bias by removing pairs of reads with the same insert length.

Three datasets were generated following the *de novo* assembly process, one for both river systems combined, and two to analyze the Volga and Meramec River data independently. For each of the datasets, the ‘*‐r −0.80*’ flag was implemented for the *populations* command (*STACKS*) as an optimization target and filtering step to ensure only SNP loci shared by a minimum of 80% of all individuals in a population are retained (Rochette & Catchen, 2017). The ‘‐‐*write*‐*single*‐*snp*’ program flag was additionally applied to restrict the analysis to the first SNP per locus and lessen the potential for error created by linkage disequilibrium. Finally, a minor allele frequency cutoff of 5% was applied with ‘‐*min*‐*maf 0*.*05*’ to filter and remove potential outlier loci. Supplementary program options for the *populations* function were used concurrently to generate values for differentiation among sampling localities in expected and observed levels of heterozygosity, and inbreeding coefficients (Rochette & Catchen, 2017). The software package *PGDSpider 2*.*1*.*1*.*5* was used for additional conversion and formatting of the *populations* output (Lischer & Excoffier, 2012).

### 2.3 Population genomics

To identify and characterize genetic clusters within the combined river dataset, and within the river‐only datasets, two methods were used for comparison: a Bayesian assignment test and a discriminant analysis of principal components (DAPC). The Bayesian assignment test was performed using the software package *fastStructure* (Raj et al., 2014). DAPC analyses were run using the *adegenet* package for R (Jombart, 2008; Jombart et al., 2010).

Large modern SNP datasets can impose challenging computational requirements in time and processing power. The software package *fastStructure* makes use of efficient algorithms to employ a Bayesian framework model for the inference of the total genetic clusters (K) within the data, and assignments of fish to a cluster based on individual genotypes without a priori definitions (Falush et al., 2007; Hubisz et al., 2009; Pritchard et al., 2000; Raj et al., 2014). The range of potential K values chosen included a value 1 above the total number of sampling localities within each dataset (Pritchard et al., 2000). *K* values = 1–11 were analyzed for the combined river dataset, and *K* = 1–6 for each river separately. Each *K* value was run for 500 iterations. The supplemental program *Structure_threader* was used to decrease the overall processing time required by *fastStructure* (Pina‐Martins et al., 2017). *Structure_threader* also automated the identification of the optimal number of *K* clusters for each dataset using the *chooseK*.*py* script (Raj et al., 2014). Visualizations and plotting of population memberships and admixture from *fastStructure* outputs were completed using *Distruct v*.*2*.*3* (Chhatre, 2019).

A DAPC identifies differences between groups through discriminant functions (Jombart et al., 2010). This analysis can be substantially affected by the selection of user‐defined numbers of principal components (PC) to preserve. The *find*.*clusters* and *xvalDapc* functions within the R package *adegenet* provided a procedure for effective cross‐validation and optimization to identify the number of PCs to keep for each dataset and identify the optimal number of clusters (Jombart & Collins, 2015). The number of PCs retained for each analysis was therefore selected by using the value of primary components with the lowest root mean squared error (RMSE) after 100 iterations per PC values of 1–100 for the combined dataset, and values 1–50 for each independent river system.

Pairwise *F*_ST_ values between all localities within each dataset were calculated as described in Weir and Cockerham (1984) using the *hierfstat* package for the R platform (Goudet, 2005; R Development Core Team, 2020). An analysis of isolation by distance was conducted for the Volga‐only and Meramec‐only datasets using a Mantel test. For the Meramec‐only dataset, two analyses were run, with and without the most distant locality MO5. Pairwise F_ST_ values were linearized (*F*_ST_/1 − *F*_ST_) following Rousset (1997), and river distance measures were used. The Mantel test was conducted with 100,000 replicates in the R package *ade4* (Dray & Dufour, 2007). Finally, a nested analysis of molecular variance (AMOVA) was performed for each of the three datasets to further determine the spatial structure of genetic diversity. The analyses were performed using *Arlequin v*.*3*.*5*.*2*.*2* (Excoffier & Lischer, 2010).

## 3 RESULTS

### 3.1 Sequencing quality, SNP loci, and genetic diversity

Over 817 million 70 bp reads were generated, with an average of 8,171,812 reads per individual after demultiplexing and length trimming. Two individuals from the Volga River V01 sampling locality were removed due to low sequencing coverage. The coverage for all remaining individuals ranged from 10.61× to 20.53×. A total of 6,555,128 loci were retained for genotyping, resulting in 1,405,998 variant sites among all individuals.

The combined river dataset, which incorporated all 10 sampling locations in both the Volga and Meramec rivers, produced a mean of 13,820 variant SNP loci per locality (Table 1A). There was a greater average number of variant loci per locality in the Meramec River as compared to the Volga River. The Meramec River localities also exhibited greater genetic diversity (*H*_O_) than the Volga River localities (Table 1A). Within the Volga‐only dataset, an average of 8374 variant SNP loci was identified among the five sampled localities (Table 1B), which was lower than what was observed in the combined river dataset, 13,433 (Table 1A). The genetic diversity for the localities based on this dataset was much higher than those observed in the combined river dataset. Locality V01 had the lowest number of variant loci and the highest estimate of genetic diversity. The Meramec‐only dataset had an average of 14,342 variant SNP loci across the five localities (Table 1C), which is similar to what was observed in the combined dataset, 14,207 (Table 1A). The diversity estimates in this dataset were also similar to what was observed in the combined dataset. Locality MO3 had the greatest number of variant loci and locality MO5 had the highest estimate of genetic diversity.

**TABLE 1:** Genetic diversity estimates for the (A) Combined river, (B) Volga‐only, and (C) Meramec‐only datasets including the number of individuals (*N*), average number of individuals per locus and standard error (*SE*), variant loci, observed heterozygosity (*H*_O_) and standard error (*SE*), expected heterozygosity (*H*_E_) and standard error (*SE*), and inbreeding coefficients (*F*_IS_) and standard error (*SE*)

River (Site ID)	*N*	Avg. *N*/locus (*SE*)	Variant loci	*H*_O_ (*SE*)	*H*_E_ (*SE*)	*F*_IS_ (*SE*)
(A) Combined River						
Volga (V01)	8	7.6 (0.005)	10,156	0.143 (0.002)	0.132 (0.002)	0.001 (0.005)
Volga (V02)	10	9.6 (0.006)	14,527	0.157 (0.002)	0.151 (0.002)	0.014 (0.006)
Volga (V03)	10	9.6 (0.006)	14,779	0.155 (0.002)	0.159 (0.002)	0.039 (0.006)
Volga (V04)	10	9.7 (0.005)	13,843	0.150 (0.002)	0.150 (0.002)	0.026 (0.005)
Volga (V05)	10	9.6 (0.006)	13,859	0.150 (0.002)	0.154 (0.002)	0.039 (0.006)
Volga Average	48	9.2	13,433	0.151	0.149	0.024
Meramec (M01)	10	9.7 (0.006)	14,209	0.231 (0.000)	0.227 (0.002)	0.028 (0.006)
Meramec (M02)	10	9.0 (0.006)	12,439	0.230 (0.002)	0.225 (0.002)	0.029 (0.006)
Meramec (M03)	10	9.4 (0.006)	17,485	0.228 (0.002)	0.225 (0.001)	0.035 (0.006)
Meramec (M04)	10	8.9 (0.003)	12,974	0.235 (0.002)	0.227 (0.002)	0.019 (0.003)
Meramec (M05)	10	9.6 (0.006)	13,927	0.244 (0.002)	0.230 (0.002)	0.005 (0.006)
Meramec Average	50	9.3	14,207	0.234	0.227	0.023
All Average	98	9.3	13,820	0.192	0.188	0.024

Site ID	*N*	Avg. *N* per locus (*SE*)	Variant loci	*H*_O_ (*SE*)	*H*_E_ (*SE*)	*F*_IS_ (*SE*)
(B) Volga only						
V01	8	7.6 (0.007)	5,714	0.298 (0.003)	0.277 (0.002)	0.002 (0.007)
V02	10	9.5 (0.008)	9,232	0.291 (0.002)	0.281 (0.002)	0.025 (0.008)
V03	10	9.4 (0.008)	9,534	0.283 (0.002)	0.287 (0.002)	0.066 (0.008)
V04	10	9.6 (0.008)	8,668	0.285 (0.002)	0.282 (0.002)	0.045 (0.008)
V05	10	9.5 (0.008)	8,725	0.282 (0.000)	0.288 (0.002)	0.067 (0.008)
Average	48	9.1	8,375	0.288	0.283	0.041
(C) Meramec only						
M01	10	9.7 (0.006)	14,311	0.260 (0.002)	0.255 (0.001)	0.033 (0.006)
M02	10	9.0 (0.006)	12,546	0.258 (0.002)	0.254 (0.001)	0.035 (0.006)
M03	10	9.4 (0.006)	17,776	0.252 (0.002)	0.249 (0.001)	0.041 (0.006)
M04	10	8.9 (0.003)	13,057	0.265 (0.002)	0.256 (0.001)	0.023 (0.003)
M05	10	9.6 (0.006)	14,019	0.275 (0.002)	0.260 (0.001)	0.011 (0.006)
Average	50	9.3	14,342	0.262	0.255	0.029

There was no evidence of inbreeding in any of the datasets (Table 1). The variation in the number of variant SNP loci and genetic diversity estimates between the combined and independent river datasets is attributed to the *STACKS populations ‘*‐*r 0*.*80’* flag. The command dictated the inclusion of loci only when present within 80% of individuals and therefore was dependent on the localities specified during the generation of the datasets.

### 3.2 Population genomics

**FIGURE 3:**
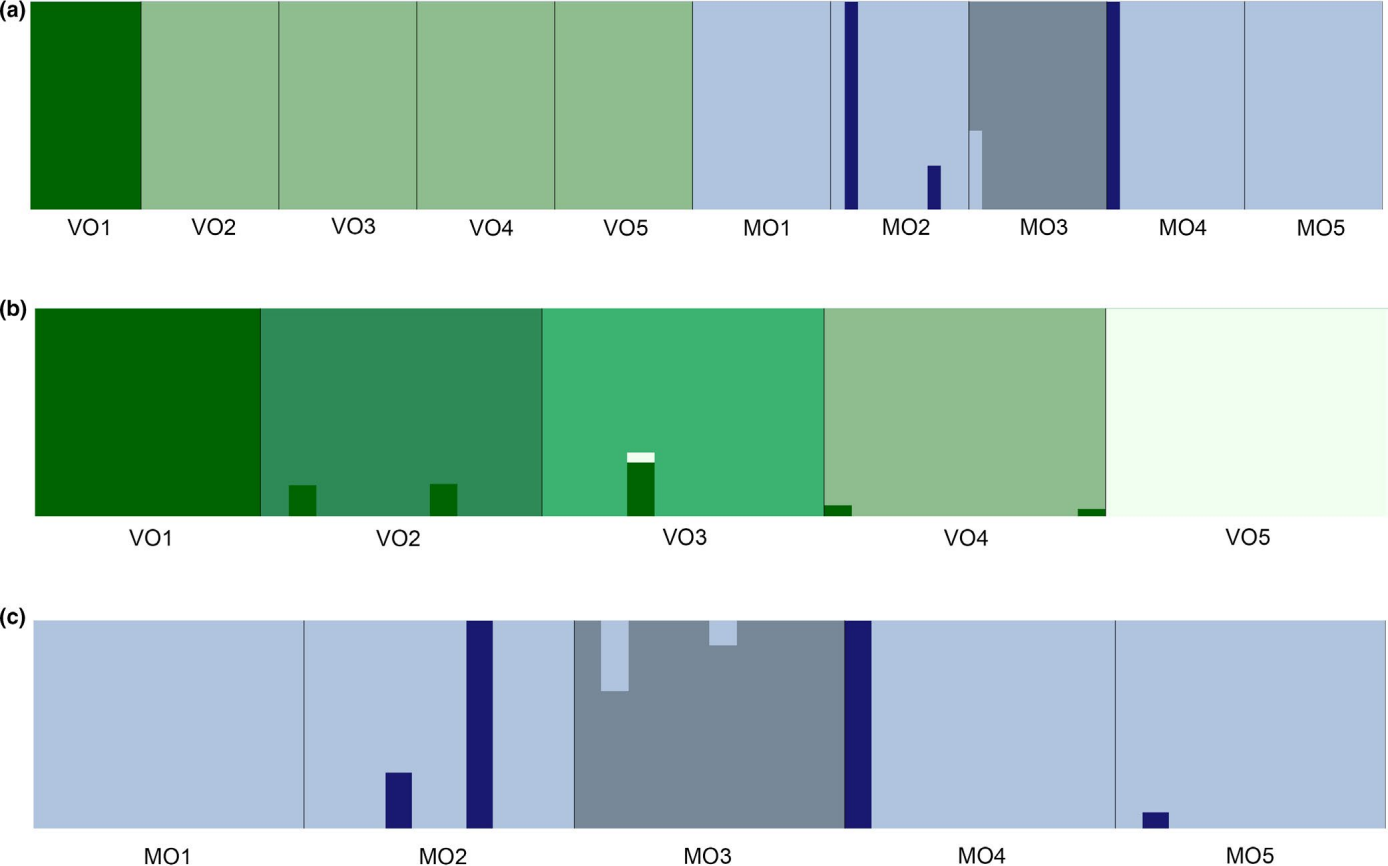
Estimated membership coefficients based on admixture analyses. Vertical axis indicates membership coefficient and the horizontal axis indicates the locality for each individual. (a) Combined river dataset for *K* = 4, (b) Volga‐only dataset for *K* = 4, and (c) Meramec‐only dataset for *K* = 3

The Bayesian assignment test identified an optimal genetic clustering value of *K* = 5 for the combined river dataset (Figure 3a). Two clusters were observed in the Volga River that are distinct from the three clusters identified in the Meramec River. Within the Volga River, localities VO2, VO3, VO4, and VO5 were contained in a single cluster with no admixed individuals. Locality VO1 represented a distinct cluster also with no admixed individuals. Within the Meramec River, the most common cluster was found in all localities with localities MO1 and MO5 entirely containing this cluster with no admixed individuals. Localities MO2 and MO4 contained individuals with a second cluster with one admixed individual in MO2. Locality MO3 contained a unique cluster with one admixed individual with the most common cluster found in the Meramec River.

The Volga‐only dataset had an optimal genetic cluster size of *K* = 5 (Figure 3b). A greater amount of population substructure was observed in this dataset as compared to the combined river dataset (Figure 3a), with each of the localities representing a distinct cluster. Localities VO1 and VO5 represented unique clusters with no admixed individuals. Localities VO2 and VO4 contained individuals with admixture from the cluster in locality VO1. Locality VO3 had a single individual with admixture from the clusters in localities VO1 and VO5. The Meramec‐only dataset had an optimal genetic cluster size of *K* = 3 (Figure 3c) and the pattern observed was very similar to what was observed in the combined dataset.

**FIGURE 4:**
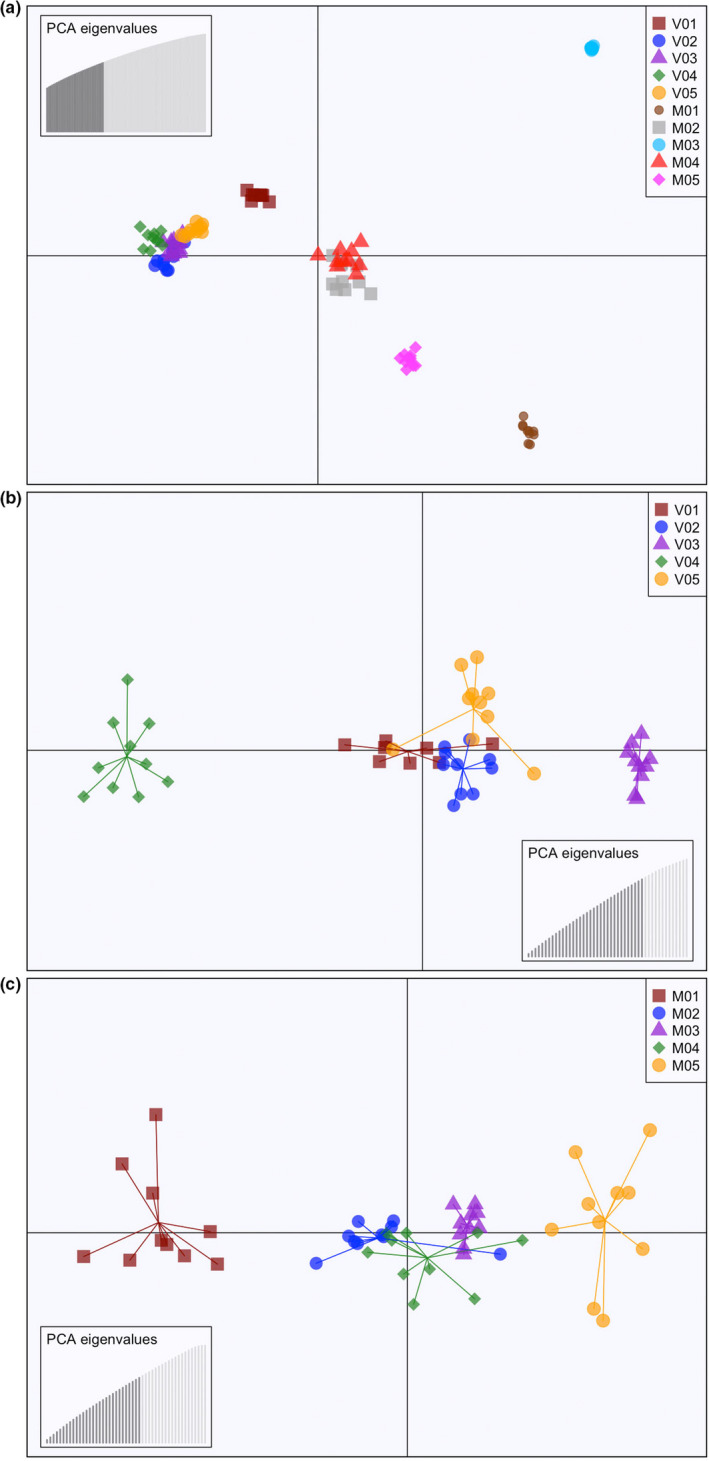
Scatterplots from discriminant analysis of principal components (DAPC) with individuals designated by locality. Eigenvalues of the analyses are displayed as insets. (a) Combined river dataset, *K* = 5. (b) Volga‐only dataset, *K* = 5. (c) Meramec‐only dataset, *K* = 3

The DAPC cross‐validation functions determined PC counts with the lowest RMSE to be PC = 30 for the combined river, PC = 35 for the Volga‐only, and PC = 25 for the Meramec‐only datasets. The DAPC conducted for the combined river dataset had BIC scores lowest for *K* = 5. This is consistent to what was observed in the Bayesian assignment test and supports a genetic distinction between the Volga and Meramec River populations (Figure 4a). The Meramec River localities displayed a higher amount of distinct genetic clustering than observed within the Volga River. Within the Meramec, locality MO3 was the most distinct and localities MO2 and MO4 were the most similar and overlapping. Localities MO5 and MO1 also formed distinct clusters. Within the Volga River, localities VO2, VO3, VO4, and VO5 were closely associated and overlapping. Locality VO1 was the most distinct. The results of the DAPC for the Volga‐only dataset had BIC scores lowest for *K* = 5. This again was consistent with the Bayesian assignment test and revealed greater population substructure with localities VO4 and VO3 forming distinct clusters on opposite ends of the plot (Figure 4b). Individuals in localities VO1, VO2, and VO5 were closely associated with some overlap. The results of the DAPC for the Meramec‐only dataset had BIC scores lowest for *K* = 3. This was also consistent with the Bayesian assignment test and revealed greater substructure (Figure 4c). The localities were arranged in the scatterplot in an upstream to downstream order with localities MO2, MO3, and MO4 overlapping in the center and localities MO1 and MO5 forming distinct clusters.

Measures of the pairwise *F*_ST_ (WC84) among all 10 localities in the combined river dataset showed a substantial amount of genetic distance between the Meramec and Volga River localities with values ranging between 0.3453 and 0.4345 and an average of 0.4083 (Table S2). However, the variance between localities within each river system was small but similar in both systems. The range of values in the Volga River was 0.0008–0.0049 with an average of 0.0028, and the range of values in the Meramec River was 0.0003–0.0063 with an average of 0.0023. In the river‐only datasets, the pairwise F_ST_ values were small and nearly identical to the observations in the combined river dataset (Table S3).

**FIGURE 5:**
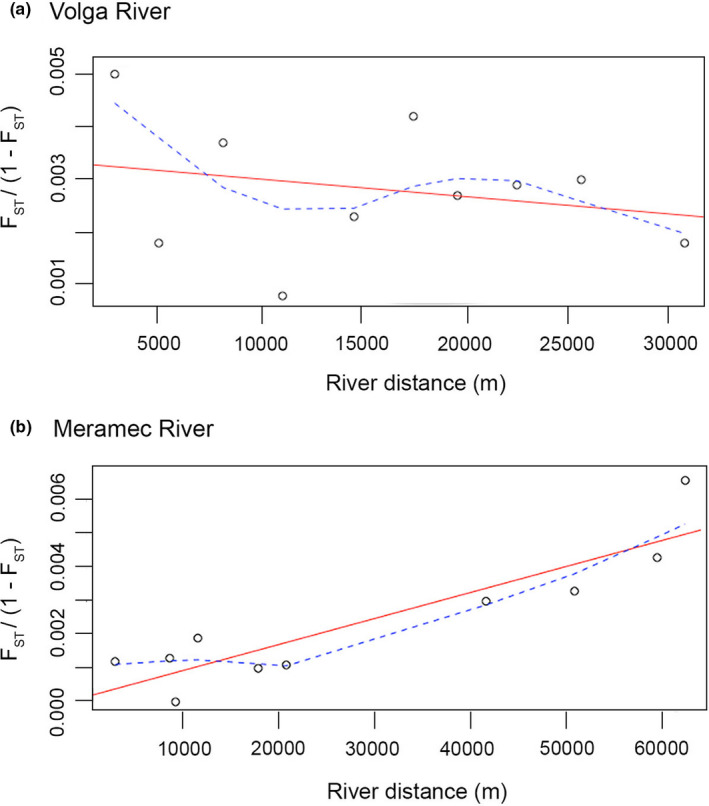
Scatter plots of the Mantel tests for isolation by distance using in‐river distances with the regression line (red) and Lowess smoothed regression / trend line (dashed blue). (a) Volga‐only dataset (*p* = .774, *R* ^2^ = .243) and (b) Meramec‐only dataset (*p* = .017, *R* ^2^ = .899)

For the Volga‐only dataset, there was no evidence of IBD with no significant correlation between in‐river distances and linearized *F*_ST_ values (*p* = .774, *R* ^2^ = .243; Figure 5a). However, for the Meramec‐only dataset, there was evidence of IBD. There was a significant correlation between in‐river distances and linearized *F*_ST_ values (*p* = .017, *R* ^2^ = .899; Figure 5b). However, when locality MO5 was removed, there was no longer a significant correlation with *p* = .046 and *R* ^2^ = .025, graph not shown. The results of the AMOVA test on the combined river dataset revealed the genetic variation observed is explained by both differences among river systems and differences among individuals within the same locality (Table 2A). The AMOVA tests on the independent river system datasets also revealed that very little of the variation is explained by differences between localities. Most of the variation is explained by differences among individuals within localities (Table 2B, C).

**TABLE 2:** Results of the analysis of molecular variance (AMOVA)

Source of variation	*df*	Sum of squares	Variance component	% variation	Fixation indices
(A) Combined river dataset					
Among rivers	1	30,153.202	Va = 487.29756	53.26	*F*_CT_ = 0.53261
Among localities within rivers	8	2,342.309	Vb = 1.70804	0.16	*F*_SC_ = 0.00345
Within localities	186	49,444.053	Vc = 556.59940	46.58	*F*_ST_ = 0.53423
(B) Volga‐only dataset					
Among localities	4	1,648.328	Va = 0.87886	0.22	
Within localities	91	35,207.832	Vb = 395.47041	99.78	*F*_ST_ = 0.00222
(C) Meramec‐only dataset					
Among localities	4	2,734.230	Va = 1.73067	0.35	
Within localities	95	57,768.503	Vb = 805.75158	99.65	*F*_ST_ = 0.00352

## 4 DISCUSSION

The results of this study support the prediction that populations in glaciated drainages in the North do not fit expectations under the stream hierarchy model, whereas populations in the unglaciated South do. The pattern observed within the Volga River in the upper Mississippi River basin reflects either recurrent extirpation or recolonization of *E*. *caeruleum* into the region caused by a disruption in the riverscape by repeated glacial advance and retreat and contemporary fragmentation of populations in a highly impacted environment. In contrast, the pattern observed within the Meramec River in the Ozark Plateau strongly adhered to the stream hierarchy model. This region was not directly impacted by glacial advances and the age and relative stability of the habitat and drainage patterns allowed for populations of *E*. *caeruleum* to persist uninterrupted. The observed genetic signature of the southern population was shaped by the life history and habitat preferences of the species rather than cycles of geologic and climatic disturbance or fragmentation of populations.

### 4.1 Colonization of the upper Mississippi River basin

Analyses of the combined river dataset revealed a signature of expansion of populations into the glaciated riverscape of the upper Mississippi River basin. Even though the study sites were chosen for comparison due to their similarity in habitat and lack of obvious barriers to dispersal, they revealed different patterns of spatial genetic structure. The northern population in the Volga River (Figure 2) exhibited low genetic diversity (Table 1A) and little population substructure (Figures 3a, 4a). In contrast, the southern Ozark Plateau population in the Meramec River exhibited greater genetic diversity and increased population substructure. The northern and southern populations are also genetically distinct with no evidence of shared clusters (Figures 3a, 4a). Both the AMOVA (Table 2A) and *F*_ST_ (Table S2) results indicated the greatest sources of genetic variation are between the northern and southern populations.

The conclusion of expansion to the North from a southern refugium in the Ozark Plateau following glacial retreat is consistent with a range‐wide phylogeographic study of *E*. *caeruleum* based on mtDNA sequence data (Ray et al., 2006). This pattern has also been repeatedly observed in other fishes within the region that also exhibit a similar disjunct distribution west of the Mississippi River. Phylogeographic studies of the Ozark minnow (*Notropis nubilus*; Berendzen et al., 2010), gilt darter (*Percina evides*; Near et al., 2001), Carmine shiner (*Notropis percobromus*; Berendzen et al., 2008), and northern hogsucker (*Hypentelium nigricans*; Berendzen et al., 2003) revealed shallow genetic divergences and lack of geographic structure between the northern and southern populations. It is hypothesized that populations of fishes expanded northward either during the Sangamon interglacial period between ~125,000 and 75,000 years ago or following the last glacial maximum ~19,000 to 10,500 years ago (Berendzen et al., 2010; Near et al., 2001).

This repeated pattern supports the influence of periodic glaciation during the Quaternary Period on shaping the distribution of the contemporary fish assemblage in the upper Mississippi River basin. As ice sheets retreated, populations of freshwater fishes expanded northward out of an Ozarkian refugium into suitable habitat in the upper Mississippi River basin (Berendzen et al., 2010; Burr & Page, 1986). Following colonization of the region, contemporary populations in the North became isolated from Ozark Plateau due to the loss of suitable habitat in the intervening region. The aquatic habitat and flow regime of river systems flowing through southern Iowa and northern Missouri were altered during the mid‐Pleistocene interglacial periods between the Pre‐Illinoian and Illinoian Glacial Stages, ~500,000–300,000 years ago (Anderson, 1998; Bettis, 1989; Elfrink & Siemens, 1998). As the ice sheet retreated, immense volumes of glacial melt carried large quantities of fine‐grained sediments to the alluvial plains adjacent the Mississippi River, which led to extensive Aeolian loess accumulation throughout the Southern Iowa and Northern Missouri Drift Plain (Anderson, 1998; Forman et al., 1992; Muhs et al., 2018; Pflieger, 1975; Young & Hammer, 2000). This resulted in changes to the clarity and siltation of rivers in this region and eliminated habitat for *E*. *caeruleum* and other species with similar preferences.

### 4.2 Historical range contraction and expansion

Analyses of the Volga River‐only dataset revealed a spatial distribution of genetic diversity that does not fit expectations under the stream hierarchy model and is not consistent with the life history of the rainbow darter. In contrast to the combined river dataset, the Volga‐only dataset revealed a greater number of genetic clusters with each locality representing a unique cluster (Figure 3b). However, there was no evidence of isolation by distance (Figure 5a). The F_ST_ values (Table S3A) indicated that the two most distant localities VO1 and VO5 are more similar than the two neighboring localities VO3 and VO4 (Figure 2). This is supported by the pattern observed in the DAPC (Figure 4b).

There are two possible explanations for the spatial genetic pattern observed in the Volga River: fragmentation of local populations due to isolation or historical range contractions and expansion or the contemporary influence of human activity. The Volga River flows through a highly impacted landscape that has been dramatically altered by intensive agricultural activities and urbanization (Figure 2). Rivers and streams have been heavily impacted, causing extensive modification to habitat structure, water quality, and flow regime (Knox, 2007; Menzel, 1983). However, given the life history of rainbow darter and the distribution of localities within the system, the observed spatial pattern of genetic diversity does not fit expectations for fragmentation and isolation. Given the short geological timeframe of contemporary impacts, it would be predicted that neighboring localities would be more genetically similar. However, this was not observed.

The best explanation is this pattern results from the influence of historical range contractions and expansions during periodic glacial cycles. In the combined river dataset, the genetic diversity in the Volga River is lower as compared to the population in the South (Table 1A). In addition, there are a fewer number of variant loci observed in the Volga‐only dataset as compared to the Meramec‐only dataset, even though they have similar heterozygosities (Table 1B,C). These observations suggest that the population in the Volga River is not in equilibrium; the observed genetic signature is not reflective of the contemporary demographic and evolutionary processes (Epps & Keyghobadi, 2015; Whitlock & McCauley, 1990). Rather, the pattern observed is a function of the founding events that established populations in the North. The bottleneck caused by the founder effect reduces variation, but can have little effect on the observed heterozygosity (Allendorf & Luikart, 2007). This is especially true when a population returns to a large size relatively quickly, which is consistent with the life history of *E*. *caeruleum*. Even though populations can be isolated, the rainbow darter tends to be very abundant and one of the most common fish in a stream (Page, 2000).

The disconnect between the contemporary landscape and the observed genetic pattern is the result of a time lag (Epps & Keyghobadi, 2015). Even though it has been 10,500 years since the retreat of the last glacial maximum, a genetic signature of expansion persists. Following a major population disturbance, it takes time for genetic variation to reach equilibrium, which is determined by a number of factors including generation time, dispersal rates, effective population size, and population growth rates (Epps & Keyghobadi, 2015). Based on the complex interactions of these factors, it can take tens of thousands of generations for a population to reach equilibrium (Varvio et al., 1986; Zellmer & Knowles, 2009). This conclusion is further supported by the results of the analyses of the Meramec River‐only dataset; see Discussion below.

The interpretation of the observed genetic signature in the Volga River‐only dataset is consistent with other landscape genetic studies of *E*. *caeruleum*. A recent study utilized microsatellite data to understand the spatial distribution of genetic diversity of the rainbow darter in tributaries of the upper Mississippi River basin in northeast Iowa (Davis et al., 2015). The analyses revealed a single genetic population and no evidence of significant population subdivision despite the large geographic distance separating local populations. The population of rainbow darters in this region was genetically diverse, but the diversity was evenly distributed across the landscape suggesting extensive gene flow and connectivity among population across the landscape. After considering the life history and habitat requirements of the rainbow darter and the glacial history of the region, they concluded that the best explanation was historical events overwhelmed the observed genetic signature and the data were unable to detect the influence of contemporary processes (Davis et al., 2015). Although this study came to a similar conclusion, the use of microsatellite DNA markers presumably limited the resolution of the genetic data. The genome‐wide SNP data utilized in this study were able to reveal fine‐scale population substructure that was not observed in Davis et al. (2015). Haponski et al. (2009) observed similar patterns of genetic variation in *E*. *caeruleum* populations in glaciated regions of the Lake Erie and the Ohio River basins east of the Mississippi River.

### 4.3 Adherence to the stream hierarchy model

In stark contrast, the analyses of the Meramec River‐only dataset revealed a spatial distribution of genetic diversity that is structured in an upstream‐to‐downstream pattern that corresponds to the stream hierarchy model. There was evidence of a strong correlation of genetic differentiation with river distance (Figure 5b), which was not observed when the most distant locality, MO5, was removed. However, this was further supported by the pattern observed in the DAPC (Figure 4c) and the pairwise F_ST_ values (Table S3B). There is greater genetic diversity (Table 1C) observed in the Meramec River than in the northern Volga River, which is best explained by differences among individuals within populations (Table 2C). There were also fewer genetic clusters observed (Figures 3c, 4c), suggesting greater connectivity among the local populations.

The adherence to the stream hierarchy model observed in the Meramec River‐only dataset suggests that habitat in the region remained relatively undisturbed by the climatic changes in the Quaternary period. The river valleys of the Ozark Plateau are older and entrenched, and lack sedimentation levels found within the glacial drift plains in the North (Forman et al., 1992; Galloway et al., 2011). The lotic habitat of these rivers also contains nearly continuous suitable habitat for the rainbow darter. Taking these factors together, the best explanation for the pattern observed is that life history of *E*. *caeruleum*, including high site fidelity and diet preferences of the species, shaped the spatial distribution of genetic diversity. Rainbow darters and their preferred prey have a preference for fast‐moving riffle habitats and are less prevalent in deeper slow‐moving pools (Hicks & Servos, 2017; Mueller et al., 2020). Therefore, the riffle–run–pool structure of streams may represent a permeable barrier to migratory movement further contributing to the observed pattern.

## 5 CONCLUSIONS

The results of this study highlight the importance for understanding the geologic and climatic history of a region and the life history of an organism when interpreting population genomic patterns. The same species in different environments can have very different patterns of genetic diversity due to the dynamic nature of the landscape. This is especially true in riverine environments. In riverscapes, where aquatic habitats have been stable over long periods of time, the spatial distribution of genetic diversity will likely fit expectations of models based on the life history of the organism and patterns of the drainage network. However, in riverscapes that are impacted, the observed patterns may not be obvious and are often difficult to interpret based on contemporary processes. In addition, these genetic signatures may persist for a long period of time due to the effects of a time lag.

## CONFLICT OF INTEREST

None declared.

## AUTHOR CONTRIBUTION

**Jon M. Luiken:** Conceptualization (equal); Data curation (equal); Formal analysis (lead); Writing – original draft (lead). **Tony Gamble:** Data curation (supporting); Formal analysis (supporting); Writing – original draft (supporting). **Peter B. Berendzen:** Conceptualization (lead); Data curation (equal); Formal analysis (lead); Funding acquisition (lead); Writing – original draft (equal).

## Supporting information

Table S1

Table S2

Table S3

## Data Availability

All three datasets were uploaded to the Dryad Digital Repository (https://doi.org/10.5061/dryad.6m905qg17). The sequence data were deposited in the NCBI SRA database under BioProject ID PRJNA781970.
