# Supplementary material for: Comparative riverscape genomics of the rainbow darter (Etheostoma caeruleum) in glaciated and unglaciated environments
Source: Ecol Evol. 2021 Dec 1;11(24):18305–18. doi: 10.1002/ece3.8422 (PMC8717317; doi:10.1002/ece3.8422)
Supplement: Supplementary file 1 — Table S1 [file ECE3-11-18305-s002.docx]

**Table S1**: Sampling ID, coordinates, and voucher number for each locality included in this study. JFBM = Bell Museum of Natural History Fish collection, University of Minnesota.

| **Locality ID** | **Latitude (N.)** | **Longitude (W.)** | **Voucher Number** |
| --- | --- | --- | --- |
| Volga River |  |  |  |
| V01 | 42.78801 | -91.88205 | JFBM 49824 |
| V02 | 42.81858 | -91.87847 | JFBM 49823 |
| V03 | 42.84436 | -91.79318 | JFBM 49822 |
| V04 | 42.83861 | -91.77200 | JFBM 49825 |
| V05 | 42.86248 | -91.76365 | JFBM 49821 |
| Meramec River |  |  |  |
| M01 | 37.63617 | -91.41185 | JFBM 49829 |
| M02 | 37.65924 | -91.41496 | JFBM 49830 |
| M03 | 37.70125 | -91.44697 | JFBM 49831 |
| M04 | 37.74572 | -91.43385 | JFBM 49832 |
| M05 | 37.95130 | -91.50868 | JFBM 49828 |
