# Supplementary material for: Comparative riverscape genomics of the rainbow darter (Etheostoma caeruleum) in glaciated and unglaciated environments
Source: Ecol Evol. 2021 Dec 1;11(24):18305–18. doi: 10.1002/ece3.8422 (PMC8717317; doi:10.1002/ece3.8422)
Supplement: Supplementary file 2 — Table S2 [file ECE3-11-18305-s003.docx]

**Table S2** Pairwise F_ST_ (WC84) estimates of genetic distance between localities for the combined river dataset.

|  | **V01** | **V02** | **V03** | **V04** | **V05** | **M01** | **M02** | **M03** | **M04** | **M05** |
| --- | --- | --- | --- | --- | --- | --- | --- | --- | --- | --- |
| **V01** | ■ |  |  |  |  |  |  |  |  |  |
| **V02** | 0.0019 | ■ |  |  |  |  |  |  |  |  |
| **V03** | 0.0026 | 0.0022 | ■ |  |  |  |  |  |  |  |
| **V04** | 0.0030 | 0.0040 | 0.0049 | ■ |  |  |  |  |  |  |
| **V05** | 0.0016 | 0.0026 | 0.0008 | 0.0041 | ■ |  |  |  |  |  |
| **M01** | 0.3954 | 0.4185 | 0.4050 | 0.4242 | 0.4144 | ■ |  |  |  |  |
| **M02** | 0.4113 | 0.4196 | 0.4074 | 0.4299 | 0.4194 | 0.0016 | ■ |  |  |  |
| **M03** | 0.3453 | 0.3852 | 0.3722 | 0.3829 | 0.3751 | 0.0017 | 0.0011 | ■ |  |  |
| **M04** | 0.4133 | 0.4273 | 0.4119 | 0.4345 | 0.4246 | 0.0012 | 0.0012 | 0.0001 | ■ |  |
| **M05** | 0.4006 | 0.4247 | 0.4119 | 0.4309 | 0.4212 | 0.0063 | 0.0043 | 0.0031 | 0.0025 | ■ |
