# Supplementary material for: Comparative riverscape genomics of the rainbow darter (Etheostoma caeruleum) in glaciated and unglaciated environments
Source: Ecol Evol. 2021 Dec 1;11(24):18305–18. doi: 10.1002/ece3.8422 (PMC8717317; doi:10.1002/ece3.8422)
Supplement: Supplementary file 3 — Table S3 [file ECE3-11-18305-s001.docx]

**Table S3** Pairwise F_ST_ (WC84) estimates of genetic distance below the diagonals with measures of the river distance (km) between localities above the diagonals. A. Volga-only dataset. B. Meramec-only dataset.

| 1. **Volga-only dataset** | | | | | |
| --- | --- | --- | --- | --- | --- |
|  | **V01** | **V02** | **V03** | **V04** | **V05** |
| **V01** | ■ | 5.05 | 19.63 | 22.53 | 30.72 |
| **V02** | 0.0018 | ■ | 14.58 | 17.48 | 25.67 |
| **V03** | 0.0027 | 0.0023 | ■ | 2.9 | 11.09 |
| **V04** | 0.0029 | 0.0042 | 0.0050 | ■ | 8.19 |
| **V05** | 0.0018 | 0.0030 | 0.0008 | 0.0037 | ■ |
| 1. **Meramec-only dataset** | | | | | |
|  | **M01** | **M02** | **M03** | **M04** | **M05** |
| **M01** | ■ | 2.93 | 11.57 | 20.81 | 62.49 |
| **M02** | 0.0012 | ■ | 8.64 | 17.88 | 59.56 |
| **M03** | 0.0019 | 0.0013 | ■ | 9.24 | 50.92 |
| **M04** | 0.0011 | 0.0010 | 0.0000 | ■ | 41.68 |
| **M05** | 0.0066 | 0.0043 | 0.0033 | 0.0030 | ■ |
